# Modulation of frontal effective connectivity during speech

**DOI:** 10.1016/j.neuroimage.2016.01.037

**Published:** 2016-10-15

**Authors:** Rachel Holland, Alex P. Leff, William D. Penny, John C. Rothwell, Jenny Crinion

**Affiliations:** a Institute of Cognitive Neuroscience, University College London, 17 Queen Square, London WC1N 3AR, UK; b Wellcome Trust Centre for Neuroimaging, University College London, 12 Queen Square, London WC1N 3BG, UK; c Human Movement and Balance Unit, Institute of Neurology, 33 Queen Square, London WC1N 3BG, UK; d Language and Communication Sciences, City University London, Northampton Square, London EC1R 0JD, UK

**Keywords:** DCM — Dynamic Causal Modelling, tDCS — transcranial direct current stimulation, fMRI — functional magnetic resonance imaging, IFS — inferior frontal sulcus, VPM — ventral premotor cortex, Speech production

## Abstract

Noninvasive neurostimulation methods such as transcranial direct current stimulation (tDCS) can elicit long-lasting, polarity-dependent changes in neocortical excitability. In a previous concurrent tDCS-fMRI study of overt picture naming, we reported significant behavioural and regionally specific neural facilitation effects in left inferior frontal cortex (IFC) with anodal tDCS applied to left frontal cortex (Holland et al., 2011). Although distributed connectivity effects of anodal tDCS have been modelled at rest, the mechanism by which ‘on-line’ tDCS may modulate neuronal connectivity during a task-state remains unclear. Here, we used Dynamic Causal Modelling (DCM) to determine: (i) how neural connectivity within the frontal speech network is modulated during anodal tDCS; and, (ii) how individual variability in behavioural response to anodal tDCS relates to changes in effective connectivity strength. Results showed that compared to sham, anodal tDCS elicited stronger feedback from inferior frontal sulcus (IFS) to ventral premotor (VPM) accompanied by weaker self-connections within VPM, consistent with processes of neuronal adaptation. During anodal tDCS individual variability in the feedforward connection strength from IFS to VPM positively correlated with the degree of facilitation in naming behaviour. These results provide an essential step towards understanding the mechanism of ‘online’ tDCS paired with a cognitive task. They also identify left IFS as a ‘top-down’ hub and driver for speech change.

## Introduction

Transcranial direct current stimulation (tDCS) is a non-invasive brain stimulation method, which can be used to modulate spontaneous cortical activity in the human brain in a polarity-dependent way (Nitsche and Paulus, 2000, Nitsche and Paulus, 2001). Increasingly, the method is used as a therapeutic tool (Hummel et al., 2005, Boggio et al., 2008, Baker et al., 2010). Recent functional neuroimaging studies have investigated how changes in connectivity within resting-state networks are related to stimulation. For example, anodal tDCS, thought to increase cortical excitability, has been shown to alter connectivity within large-scale functional networks when delivered either before (Keeser et al., 2011, Pena-Gomez et al., 2011, Polania et al., 2011, Pereira et al., 2012, Polania et al., 2012) or during resting-state functional magnetic resonance imaging (fMRI) (Meinzer et al., 2012). However, the mechanism by which an externally applied field may interact and modulate neuronal activity during a given cognitive task-state and how it relates to changes in behaviour has yet to be determined. The present study is unique in this regard. Here, we used Dynamic Causal Modelling (DCM) to explore changes in effective connectivity during a concurrent tDCS-fMRI study of overt picture naming. Resulting model parameters from the DCM were used to provide a measure of both the strength and direction of neuronal interactions between pre-specified left frontal regions known to be important for speech production (Penny et al., 2004, Penny et al., 2010, Friston, 2011). Using this approach our data provide novel insights into the underlying neuronal dynamics of anodal tDCS that operate on the naming network.

In some cognitive and neurobiological models, cognitive functions are specified in distributed, inter-connected, overlapping and highly parallel processing networks (Hebb, 1949, Horwitz, 2003). This theoretical framework has been used to characterize a variety of different complex cognitive skills, including picture naming (Seidenberg and McClelland, 1989, McClelland and Rogers, 2003). From this perspective, connections within a distributed naming network can be altered and differentiated via exposure, or experience-based learning. Similarly, behavioural and neural facilitation, or priming, of naming performance can also be seen as mediated via refinement and adjustment of connections between collaborating brain regions (Buchel et al., 1999, Weiller and Rijntjes, 1999).

The neural correlate associated with learning and facilitation is neural priming, which is characterized by a decrease in focal brain activity reflecting processes of neuronal adaptation (Henson, 2003, Grill-Spector et al., 2006). Neuronal adaptation is mediated by changes in effective connectivity between and within regions of the neural network (Buchel et al., 1999, Weiller and Rijntjes, 1999, Pleger et al., 2006). Consistent with predictions of neuronal adaptation, we previously demonstrated that anodal tDCS applied to the left inferior frontal cortex during overt picture naming concurrent with fMRI had a regionally specific neural priming effect on the BOLD signal in left inferior frontal sulcus (IFS) and left ventral premotor cortex (VPM). Priming of the neural response (decrease in BOLD signal) significantly correlated with the behavioural priming of naming response times (Holland et al., 2011). This response profile suggests that anodal tDCS promotes neural efficiency during naming.

How these neural adaptation effects are mediated by changes in effective connectivity remains unclear. Considering these data in the context of a learning framework, one may predict that facilitatory tDCS effects would be mediated by changes in inter-regional connectivity affected by anodal tDCS (Weiller and Rijntjes, 1999), or in intra-regional activity via self-connections (Penny et al., 2004). To explicitly test these predictions in the present study we used DCM to determine: (i) changes in the strength and direction of neuronal coupling within and between left IFS and VPM during anodal tDCS compared to sham; and, (ii) how, at an individual level, the variability in effective connectivity values between these same two frontal regions related to variations in observed facilitation of picture naming behaviour (faster naming response times – RTs) during anodal tDCS.

## Materials and methods

### Participants

10 right-handed, healthy native speakers of English (7 females, mean age 69 years; range 62–74 years old) participated in a functional neuroimaging study of overt picture naming concurrent with anodal tDCS. All participants had normal hearing and no previous history of metallic implants, neurological or psychiatric disease. All participants were left hemisphere dominant for speech production as determined by a previous fMRI naming study. The simple main effect of anodal tDCS on the naming network in the same subjects has been reported previously (Holland et al., 2011).

### Experimental design

**Fig. 1:**
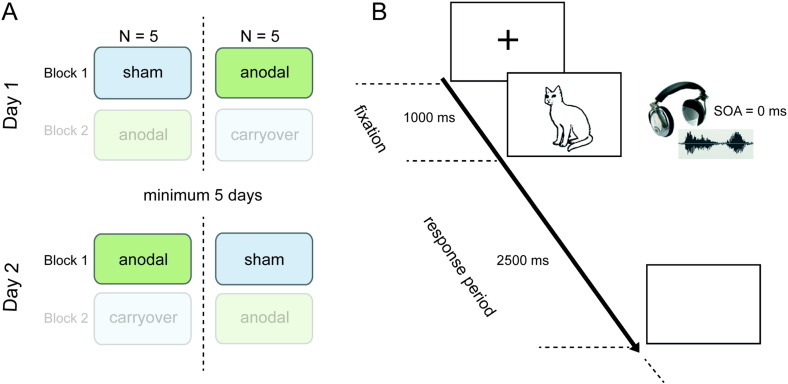
Experimental procedure. (A) Timeline of the Experimental Run Procedure. This graphically displays the two, counterbalanced orders of intervention used. Block 1 and Block 2 refer to the tDCS run order within a scanning day. For the DCM analysis, only data only from the first scanning block on each scanning day was included. (B) Timeline of the Experimental Event Procedure. A typical sequence involving presentation of a fixation cross, a picture to be named and an auditory cue in the concurrent fMRI and tDCS (anodal or sham) naming task. After a 1000 ms fixation cross pictures were displayed simultaneously with the presentation of an auditory cue (SOA = 0 ms) which was either a spoken word that matches the picture/cat/or noise control item i.e., same word spectrally rotated and noise vocoded. Each picture remained on screen for 2500 ms and participants were instructed to name aloud the object as quickly and as accurately as possible. Brain images were continuously acquired, tDCS was continuously delivered and speech responses were audio-recorded.

We targeted left frontal activity using 2 mA anodal tDCS or sham stimulation delivered for 20 min during an fMRI study of overt spoken picture naming. To avoid problems of tDCS and sham group comparability with regard to common confounding variables (e.g., age and sex) we used a within subject cross-over design where each of our 10 subjects served as his/her own control. In our previous study (Holland et al., 2011) we investigated both order and cross-modal repetition effects as each picture was presented twice across two fMRI blocks on each scanning day: once with the target's spoken name as a cue and once with an acoustic control cue (a noise-vocoded speech cue). For the DCM analysis, we were only interested in the simple main effect of anodal tDCS vs. sham during naming compared to rest. We therefore included data only from the first scanning block on each scanning day and collapsed across auditory cue types. This ensured that we avoided any potential confounds of – and interactions with – the expected behavioural and neural priming effects of practice (order) or cross-modal repetition (cues) which would be associated with repeated exposure of items to be named on each scanning day. See Fig. 1 for a visualization of the study and task design. Full details of stimuli used and experimental procedures have been reported previously (Holland et al., 2011).

On their first scanning day, during their first naming block half the participants (N = 5) received sham stimulation. On their second scanning day, the order of intervention was reversed i.e., they received an A-tDCS naming block first. The remaining five participants had the opposite order of intervention across scanning days. Using this sequencing, the order of intervention was fully counterbalanced across participants and scanning days. A minimum of 5 and maximum of 7 days separated the two scanning days. This approach permitted measurement of both the behavioural and neural consequences of anodal tDCS during: (i) real anodal tDCS, and (ii) sham stimulation. Fig. 1A displays the run procedure.

The order of stimuli was pseudo-randomized. In their first scanning day, during their first naming block, participants saw each of the 107 high frequency monosyllabic pictures to name paired with either a word or noise cue. Participants then saw the same 107 pictures paired with the remaining cue type in the second scanning day's first naming block. The order of pictures and accompanying cues was counterbalanced both within and across participants to ensure that the same picture and cue pairing was not presented during the second scanning session. Visual stimulation was via rear video projection (JVC SX-21, Japan) and auditory stimulation by MRI compatible electrodynamic headphones (Confon, Germany). Each picture was preceded by a fixation cross for 1000 ms and displayed for 2500 ms. Auditory cues were presented simultaneously with each picture (SOA = 0 ms). Trials were presented in short blocks of 6 stimuli, separated by a fixation-only rest period of 7 s. The inter-trial interval was set to 3920 ms so as to jitter the onset on each trial across acquired volumes. Fig. 1B displays the event procedure.

### tDCS stimulation and concurrent fMRI

Anodal tDCS was generated by a specially designed MRI compatible Neuroconn stimulator system (Rogue Resolutions) and delivered at 2 mA continuously for 20 min via a pair of identical, MRI-compatible leads and rectangular rubber MRI compatible electrodes (5 × 7 cm) allowing for a current density of 0.057 mA/cm^2^. For all participants, the anode was placed over left IFC (equivalent to electrode position FC5 in a 10–10 EEG nomenclature) with the cathode placed over contralateral fronto polar cortex.

A scanner pulse triggered the onset of the stimulation at a given slice in the acquisition sequence. A 15 s “ramp-up” phase with a further 15 s of stimulation was delivered at the thresholded level prior to the onset of the first picture. A constant direct current (2 mA) was delivered for 20 min. For sham stimulation, the ramp-up phase was followed by 15 s of stimulation prior to the onset of the first picture, which was immediately followed by a 1 s ramp-down phase.

Both anodal tDCS and sham stimulation protocols produced sensations of comparable quality (a mild tingling, typically under the electrode placed over the contralateral orbital ridge). No adverse sensations, phosphenes or analogous effects were reported. Participants did detect a difference in sensations between scanning sessions (p = 0.07). However, self-reports indicated that if a difference was detected, participants could not reliably identify which was anodal tDCS. The position of the anode and cathode for each subject was recorded and reproduced across both scanning days.

### Behavioural data

Participants were instructed to name the picture as quickly and as accurately as possible. They were informed that they would hear either a word or noise cue accompanying each picture, but they were not to wait for this cue to finish before naming the picture. Overt spoken responses were recorded in the scanner using a dual-channel, noise-cancelling fibre optical microphone system (FOMRI III http://www.optoacoustics.com). Each response was reviewed offline to verify manual recording of accuracy and used to determine trial-specific reaction times for each participant. Any naming trial resulting in an incorrect response or inappropriate activation of the voice-key was excluded from the analysis of reaction times (RTs). Trials that were greater than two standard deviations from the condition mean were also excluded resulting in a total of 5.9% of data excluded. By-subject and by-item means were calculated for anodal tDCS and sham conditions and analysed using one-tailed paired t-tests. Results were considered to be significant with an alpha level of 0.01.

### Imaging

Whole-brain imaging was performed on a 3 T Siemens TIM-Trio system (Siemens, Erlangen, Germany) at the Wellcome Trust Centre for Neuroimaging. Using a 12-channel head coil we acquired T2*-weighted echo-planar images (EPI) with BOLD contrast. Each EPI comprised 48 AC/PC-aligned axial slices with sequential ascending acquisition; slice thickness of 2 mm, 1 mm inter-slice gap and a 3 × 3 mm in-plane resolution. Volumes were acquired with a repetition time (TR) of 3360 ms per volume and the first six volumes of each session were discarded to allow for T1 equilibration effects. A total of 350 volume images (344 volumes of interest, 6 dummy scans) were acquired in two consecutive runs within each session lasting approximately 20 min. Prior to the first functional run of each scanning session, a gradient field map was acquired for each participant for later B0 field distortion correction of functional images. The same scanner and hardware were used for the acquisition of all images.

The functional data were preprocessed using Statistical Parametric Mapping software (SPM8; www.fil.ion.ucl.ac.uk/spm) running under Matlab 7.7 (MathWorks, Natick, MA). The first six volumes were discarded and all subsequent volumes from each participant were realigned and unwarped, using the first image as reference and resliced with sinc interpolation. The functional images were then spatially normalized to the standard T2* template within SPM normalization software. Functional data were spatially smoothed, with a 8 mm full-width at half-maximum isotropic Gaussian kernel to allow for residual variability after spatial normalization and to permit application of Gaussian random field theory for corrected statistical inference.

Statistical analyses were first performed in a subject-specific fashion. To remove low-frequency drifts, the data were high-pass filtered using a set of discrete cosine functions with a cut-off period of 128 s. Each condition and cue type was modelled separately as an event by convolving it with the SPM canonical haemodynamic response function (HRF). We used presentation of the picture as the onset of the event to model the naming response. Movement realignment parameters were included as covariates of no interest. The resulting stimulus-specific parameter estimates were calculated for all brain voxels using the General Linear Model. The first block of fMRI data from the two different scanning days, which equated to first exposure to either anodal tDCS or sham, were then concatenated to produce a single, 4d data structure for each subject's first-level analysis. Contrast images were computed for naming items relative to rest for whole brain analyses at the second level.

### Constructions of DCMs

**Fig. 2:**
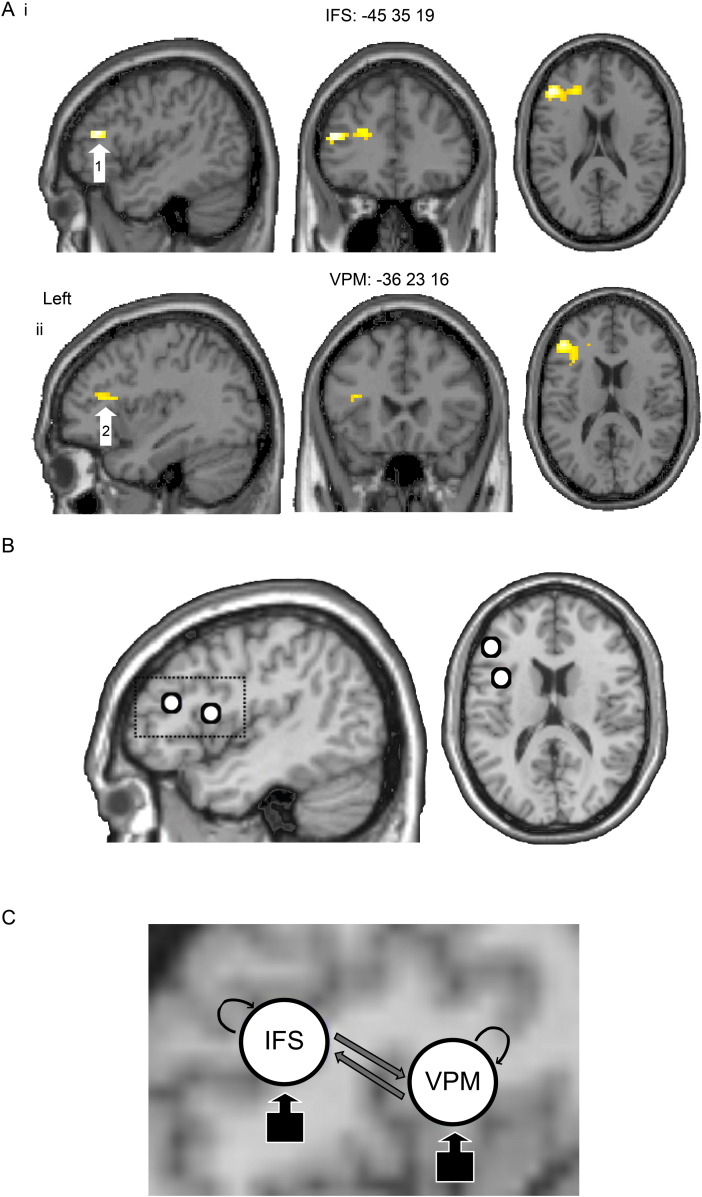
Neural effects of anodal tDCS and DCM models. (A) Statistical parametric maps showing greatest reduction in BOLD signal (uncorrected, P = 0.001) in left inferior frontal sulcus (1: IFS; z score 4.65; Ai) and ventral premotor (2: VPM; z score 3.50; Aii) as a consequence of anodal tDCS delivered during the naming task. (B) The open circles illustrate the 6 mm spherical volume of interest located at peaks of anodal tDCS effects. Regional time series data was drawn from these regions for each subject for the DCM analysis. (C) Zoom from panel B showing the fully connected Dynamic Causal Model (DCM) based on the effective connectivity of the same two regions of interest. Inputs were directed to either IFS or VPM.

Dynamic Causal Modelling (DCM) is not an exploratory technique (Friston et al., 2003). Conventional deterministic DCMs, as used here, require that specified network nodes are derived from experimental manipulation (Stephan et al., 2007). Consistent with our previous study, two left frontal regions: left IFS: -45 35 19 (Z = 4.65) and left VPM -36 23 16 (Z = 3.50), were significantly modulated by anodal tDCS vs. sham when delivered concurrently during a picture naming task and formed the basis of our DCM analysis. See Fig. 2A, i and ii for illustration of the two peaks respectively. It is common practice in DCM to restrict the analysis to only a small number of co-activated regions. At the same time it is recognized that connectivity between these regions could be mediated polysynaptically by other regions not within the model (Friston, 2011).

For model specification and estimation, we used DCM10 as implemented in SPM13A. In our analysis, the model parameters in the A matrix reflected the intrinsic connectivity between IFS and VPM (regardless of tDCS type); while the B matrix modelled any differential effect between the two tDCS types (e.g., anodal vs. sham). The C matrix coded the driving inputs into these regions (regardless of tDCS type).

A total of 6 six alternative DCMs were therefore created and subjected to Bayesian Model Selection (BMS). Variations of the model were based on inputs into either region (IFS or VPM), the presence of either feedforward, feedback connections, and/or both. Inter-regional connections (B matrix) were modelled in three different ways: IFS → VPM, IFS ← VPM and IFS ↔ VPM. Inputs were either into IFS or VPM. Crossing these two connections patterns gave six different models per subject. Self-connections were included in all B matrices (that is, they could be modulated by stimulation type) and the A matrix was ‘fully connected’ in all models. See Fig. 2C. For each participant, 6 mm spherical volumes of interest (VOI) were defined around the local or nearest supratheshold maxima (contrast: naming > rest) in IFS and VPM of the SPM[t] of each participant. Data were drawn from the same coordinate in an anodal tDCS and sham General Linear Model (GLM), using an equivalent threshold for both. In addition, we stipulated that the VOI coordinates could not exceed ± 6 mm in any direction from the target peak coordinates as illustrated in Fig. 2B. Calculation of the Euclidean distance between the coordinates for each participant also indicated that none of the VOIs overlapped spatially, (group mean = 21.61 mm, SD = 4.79). Resulting group mean coordinates for the two VOIs were: IFS (− 49 32 18), VPM (− 41 13 12).

All models were estimated for each participant using Bayesian estimation (Friston et al., 2003). The models were then ranked according to their relative posterior probability using fixed effects (FFX) Bayesian model inference (Stephan et al., 2009). Inferences about model parameters were then made using Bayesian model averaging (BMA) (Penny et al., 2004, Penny et al., 2010). If the hypotheses are framed in terms of differences at the level of connection strengths, not model architecture, then Bayesian model averaging can be applied to compute the average strengths of connections that are common to all subjects. This produces a posterior distribution over connection strengths, which is independent of model assumptions. The distribution is represented using samples. For a detailed account of the mechanics of BMA as implemented in DCM10 see (Penny et al., 2010).

In short, first, all models are estimated for each subject using Bayesian estimation (Friston et al., 2003). The models are then ranked according to their relative posterior probability, i.e., relative to the model with the poorest explanation of the observed data, and the connection values are sampled according to their posterior probability across subjects. That is, if two models have posterior probabilities of 0.1 and 0.5 for a given subject, then parameters from the second model will be sampled five times more frequently than those from the first. Ten thousand samples are selected over all subjects in a group, over models (weighted), and over connections (if a given connection is absent for a given model, then it will have a value of zero). In this way, an average model is built up that comprises the distributions of values for each connection.

**Fig. 3:**
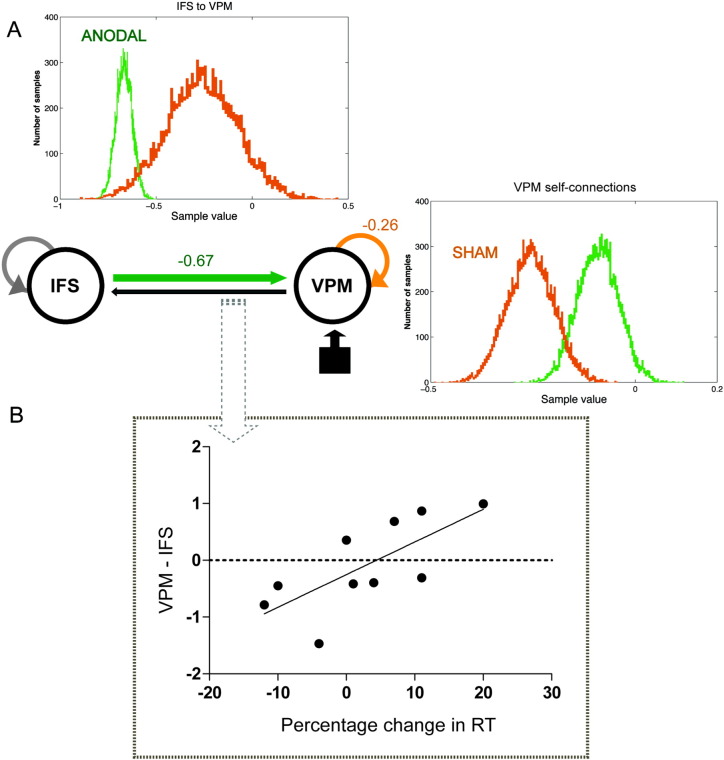
Relationship between neural connectivity and behavioural effects. (A) Connectivity results for the group are shown on the winning fully connected DCM model with inputs into the VPM. The distributions of the values of the two connections that were significantly modulated by anodal tDCS are plotted as histograms. Connection strength is plotted on the x-axis while on the y are the number of samples with these values. These 10,000 values are sampled according to their posterior probability across each subject's 6 DCMs. Connections that showed significantly different distributions for anodal tDCS vs. sham are shown in coloured arrows and plot insets: anodal (green) backward connection IFS to VPM, sham (orange) VPM self-connection. The effective connections and connection values are shown in corresponding colours on the DCM. VPM — left ventral premotor cortex; IFS — left inferior frontal sulcus. (B) The plot illustrates how individual variability in the forward connection strength (VPM to IFS) showed a significant positive correlation with naming response times (RT). Positive values on x-axis indicate faster naming RT during anodal tDCS compared to sham. Positive values on the y-axis indicate a stronger connection value during anodal tDCS compared to sham.

BMA was then used to (i) make inferences at the group level (across subjects) about differences between the two stimulation conditions (anodal tDCS vs. sham) (Fig. 3A), and (ii) to extract average connection strengths per subject per connection that were then entered into a correlational analysis (Fig. 3B). For the group analyses, we used a two-distribution test where the mean connection value for each condition (anodal tDCS and sham) was compared. Hypothesis: is the connection value different across stimulation conditions (anodal tDCS vs. sham)? If the null hypothesis of the connection strength being equivalent is refuted we can infer that the connection is modulated the stimulation condition (anodal tDCS or sham). The threshold for rejecting the null hypothesis was set to 95% probability. For the within-subject correlational analyses, DCM provides a useful tool to explore how the degree of change in effective connectivity values relate to variations in observed participants' behaviour during overt picture naming. Parameter estimates were therefore extracted from the intrinsic connectivity matrix of every participant for a between-subject correlational analysis. Here, we used a two-tailed Pearson's bivariate correlation to explore the relationship between each subject-specific DCM parameters (difference between anodal tDCS and sham connection values) and change in RT during anodal tDCS (difference in naming RTs between anodal tDCS and sham). Hypothesis: is there a correlation between change in naming RTs and connectivity values across stimulation conditions (anodal tDCS vs. sham)? The threshold for rejecting the null hypothesis was set to p < 0.05.

## Results

### Behavioural effects of anodal tDCS

Behaviourally, there was a significant effect of anodal tDCS on naming compared to sham. Naming responses remained accurate throughout and naming RTs were faster for the group during anodal tDCS (mean = 811.4 ms, SD = 94.2) compared to sham (mean = 839.5 ms, SD = 101.5). This difference did not reach significance by subjects (t(9) = 1.051, p = 0.16). However, by items, anodal tDCS elicited significantly faster naming response times (813.7 ms, SD 63.4) compared to sham (841.5, SD = 59.0) stimulation (t(106) = 6.801, p < 0.001).

### Neural connectivity effects of anodal tDCS

DCM using Bayesian Model Averaging (BMA) and a fixed effects (FFX) level of inference identified the fully inter-connected model with driving inputs to VPM was the winning model for both anodal tDCS and sham. See DCM model Fig. 3A. As often seen in empirical studies, this simulated architecture comprised positive (excitatory) forward connections (VPM to IFS) and negative (inhibitory) backward connections (IFS to VPM). Furthermore, the following connections showed significantly different distributions for anodal tDCS vs. sham: IFS backward connection to VPM (anodal 98%; sham 2%) and VPM self-connections (sham 98%; anodal 2%). As illustrated by the green (anodal tDCS) and orange (sham) effective connection arrows in Fig. 3A. There was no correlation between the Euclidean distance between the centre of the two spherical VOIs calculated for each subject and these results (VPM self-connections: r = − 0.56, df = 10, p = 0.879; VPM-IFS: r = 0.297, df = 10, p = 0.405). The remaining connections showed no significant distribution differences (IFS self-connections: anodal 69%; sham 30%; VPM forward connection to IFS: anodal 32% and sham 68%).

In addition, there was tuning of and an increase in the magnitude of the feedback connection strength from IFS to VPM during anodal tDCS (− 0.67) compared to sham (− 0.26). That this connection was more negative during anodal tDCS indicates that there was stronger backward connection between these two regions during anodal tDCS compared to sham tDCS. This is turns suggests greater inhibition. In other words, anodal tDCS increased the inhibitory effective connectivity of the IFS to VPM connection. This is illustrated by the green and orange effective connection values and distributions in Fig. 3A, top plot. In contrast, the self-connection value within the VPM was greater during sham (− 0.26) compared to anodal stimulation (− 0.09), Fig. 3A, right plot. Here, decay in the VPM self-connection value was (1/a)*log(0.5), which for sham was 2.67 s and for anodal tDCS was 7.70 s. This indicated that activity was more enduring within the region during anodal tDCS vs. sham. There was no correlation between the VPM self-connection changes and the changes in connection strength from IFS-to-VPM (r = − 0.55, df = 10, p = 0.1). The remaining connection values were not significantly modulated by tDCS: IFS self-connections (anodal − 0.57 and sham − 0.6); VPM feedforward connection to IFS (anodal 0.87 and sham 0.96).

### Relationship between neural and behavioural effects

Individual participants' behavioural facilitation (faster naming RTs) induced by anodal tDCS vs. sham varied (x-axis, Fig. 3B). A significant positive linear correlation was found between faster naming reaction time (RT) during anodal tDCS compared to sham (expressed as percentage change in anodal tDCS vs. sham) and relative DCM-derived values in the feedforward VPM to IFS connection during anodal tDCS compared to sham stimulation (r = 0.722, df = 10, p = 0.018, R^2^ = 0.52, two-tailed) (See Table 1). Removal of the subject with the lowest change in forward VPM-IFS connectivity strength (VPM-IFS connection value of − 1.5) did not affect the significance of the correlation: r = 0.748, df = 9, p = 0.02).

## Discussion

Anodal tDCS (2 mA) targeting left frontal cortices paired with a naming task resulted in more efficient naming (as indexed by faster naming RTs) without affecting subjects' accuracy. It also reduced BOLD signal in left IFS and VPM. Our DCM results suggest that anodal tDCS (i) at a group level, resulted in a more efficient naming network through a change in connectivity from IFS to VPM and VPM self-connections; and (ii) at an individual level, variability in response to anodal tDCS was reflected in variations in the feed forward connection strength from VPM to IFS that directly correlated with each subject's naming performance (RT change). A simple explanation for these data is that anodal tDCS reduced noise in the naming system making the signal (i.e. the correct word for an object e.g. /dog/) easier to detect within the noise (i.e. the competing alternative words e.g. /cat/, /animal/, /pet/). This suggests that anodal tDCS delivered over IFS is acting by a ‘top-down’ mechanism to reduce ongoing noise on IFG, filtering out irrelevant signals and thereby reducing overall VPM activity (less noise). Taken together, these data suggest Broca's area (left IFS) as a ‘top-down’ hub within the frontal cortices and driver for speech change.

(i) Neural connectivity effects of anodal tDCS
  Our DCM findings can also be interpreted within the framework of predictive coding, which is an emerging view of localisation of brain function based on context and prediction – a view that in cognitive neuroscience has increasing empirical evidence consistent with it(Brown and Brune, 2012, Koster-Hale and Saxe, 2013). In predictive coding, forward, bottom-up connections have been hypothesised to propagate prediction error signals about (unexpected) sensory information associated with the stimulus from ‘lower’ (sensory) brain areas to areas that are ‘higher’ in the cortical hierarchy(Rao and Ballard, 1999, Friston, 2010). Top-down, backward connections then send predictions based on an internal generative model about the stimulus to lower sensory areas to minimize sensory prediction error (Friston, 2010). In this context, naming with facilitatory anodal tDCS – relative to naming with sham – should result in increased predictions (selection from alternatives) and increase signal passing from higher-order IFS to lower level sensory area VPM. Compatible with this, our results showed that there was a tuning of and increase in the magnitude of the feedback connection strength from IFS to VPM during anodal tDCS. According to the predictive coding account of hierarchical processing (Friston, 2008) this indicates that online anodal tDCS increases the effect that top-down predictions play in supporting accurate picture naming.
  However, self-connection strengths within VPM also showed a shift in connectivity strength during anodal tDCS, with significantly weaker self-connections compared to sham. Within the current DCM model self-connections are constrained to be inhibitory, and they reflect the time constants of neural activation in each region. Weaker self-connections reflect more persistent regional activity. This higher background activity during anodal tDCS may effectively prime the region for the next input. In turn this may therefore reflect a relative lowering of the regional threshold in anodal tDCS compared to sham.
  Yet these two reported effects are in opposition: (a) increasing the backward IFS to VPM connection values during anodal tDCS – being an increase in inhibition – will reduce regional activity in VPM, whereas (b) reducing the self-connection value in VPM during anodal tDCS will reduce self-inhibition and increase regional activity in VPM. Given that the net effect of anodal tDCS reduced BOLD activity in VPM then (a) must be a bigger effect than (b). This indicates (i) a connectivity-specific rather than global connectivity facilitation effect of anodal tDCS in left frontal cortices and (ii) that left frontal anodal tDCS effects were most likely maximally driven by connectivity changes from higher cortical regions associated with word meaning and retrieval (Schnur et al., 2009), rather than lower-level processes of motor speech output. We speculate these predictions associated with anodal tDCS paired with naming may be the cause for improved on-line naming performance found in this study.

(ii) Relationship between individual neural and behavioural effects of anodal tDCS
  Despite our study's small sample size (n = 10) the DCM derived measures also captured the relationship between individual variability in observed behaviour and effective connectivity strengths within a task-state network. Variability in response to tDCS has been well documented in many motor cortex studies e.g., (Wiethoff et al., 2014). Our results from the group analysis identified two connections in the naming network modulated by tDCS but with no obvious behavioural correlate (all analysed trials were correct trials). The correlational analysis identified the feedforward connection from VPM to IFS that had not been identified in the group analysis. There was no simple main effect of anodal tDCS on this connection. There was however a more complex relationship here, with the numerical difference between the connection strength value during anodal tDCS compared with sham correlating with change in naming reaction time; that is, the larger the connectivity difference, the faster the subjects were to accurately name items. In predictive coding models of hierarchical inference, the role of forward connections is to transmit prediction error from earlier levels to later levels, whereas the role of backward connections is to transmit predictions from later levels to earlier levels (Rao and Ballard, 1999, Friston, 2008). Our result is compatible with anodal tDCS improving the precision with which error messages are passed forward from VPM to IFS; the quicker these errors are resolved by the neural hierarchy, the faster the reaction times should be on correctly named items.

The BOLD response is, however, too sluggish to pick out temporal effects, but EEG studies suggest that forward connectivity (error signal effects – prediction errors) are particularly important early on in peristimulus-time as they drive later, associated feedback connectivity (revised predictions) (Garrido et al., 2007). This points to another emerging dissociation between forward and backward connections: their frequency content. Several lines of evidence now suggest that forward signalling is mediated by gamma activity, or generally higher, frequencies while backward signalling is mediated by alpha/beta activity, or generally lower, frequencies (Bollimunta et al., 2011, Bosman et al., 2012, Bastos et al., 2014, Fontolan et al., 2014, van Kerkoerle et al., 2014, Bastos et al., 2015). Recently, (Bauer et al., 2014) characterized attention-dependent changes in alpha activity and gamma activity in human subjects using MEG. They found that whereas increases in alpha attentional lateralization tracked stimulus predictability, gamma attentional lateralization was suppressed by stimulus predictability. These results suggest that slower frequencies (alpha) may be used to convey predictions, while faster frequencies (gamma) could reflect prediction errors. For a review of these findings in relation to predictive coding, see (Friston et al., 2015).

While the acute neurophysiological effects of direct current (DC) stimulation are well understood, little is know about the long-term effects. One hypothesis is that DC stimulation modulates ongoing neural activity, which then translates into lasting effects via physiological plasticity. Converging with this interpretation is evidence from an in vitro animal hippocampal model of gamma oscillations and DC stimulation that shows during 10 min of stimulation, the power and frequency of gamma oscillations, as well as multiunit activity, were modulated in a polarity specific manner. Importantly, the effects on power and multiunit activity persisted for more than 10 min after stimulation terminated (Reato et al., 2015). In humans the data is less clear. Magnetic Resonance Spectroscopy (MRS) investigations have shown that anodal tDCS can modulate GABA-ergic activity (Stagg et al., 2009, Stagg et al., 2011, Kim et al., 2014). While recently it has been suggested that anodal tDCS enhances neural synchrony (Zaehle et al., 2011), including in the gamma frequency range (Antal et al., 2004). In principle, future experiments using this DCM approach in larger samples of subjects could provide a more mechanistic understanding of this anodal tDCS phenomenon, and how this might be related to the enhanced cortical communication that is thought to be facilitated by gamma frequency oscillations (Fries, 2005), and thereby might be positively correlated with effective connectivity within or between areas.

## Conclusions

Our results provide novel insight into the causal neuronal dynamics within a task-state network in response to anodal tDCS at the group and individual level. They also support the importance of the left inferior frontal cortex in naming and point to Broca's area (IFS) as a candidate site for anodal tDCS in rehabilitation protocols aiming to improve naming difficulties (anomia) in brain-damaged patients. The presentation of a concurrent cognitive task during anodal tDCS, as reported here, may be critical to maximally facilitate task-relevant cortical connectivity change. Our study is unique in this regard, as the changes in effective connectivity induced by anodal tDCS were evaluated online during an overt speech production task. The use of DCM to determine strength and directionality of neuronal interactions is an important addition to existing studies of tDCS and connectivity. We hope that the model described in this paper may allow more mechanistic questions about how anodal tDCS modulates behaviour and cortical processing and the role of effective connectivity in that to be addressed.

## Figures and Tables

**Table 1:** Relationship between neural connectivity change and behavioural effects. Effective connectivity change was correlated with naming RT facilitation during anodal tDCS vs. sham. Coefficient values for each connection value change correlated against percentage change in naming reaction time (anodal tDCS vs. sham). Bold typeface indicates significant result at the p < 0.05 level. VPM — left ventral premotor; IFS — left inferior frontal sulcus.

	VPM to IFS	VPM self-connection	IFS to VPM	IFS self-connection
Correlation coefficient value	− 0.722	− 0.290	0.205	0.468
p-value	0.018	0.416	0.571	0.172
